# Symmetric 1,1'-Dimethylferrocene-Derived Amino Acids: Their Synthesis, Characterization, Ligational and Biological Properties With Cu(II), Co(II) and Ni(II) Ions

**DOI:** 10.1155/MBD.2000.177

**Published:** 2000

**Authors:** Zahid H. Chohan

**Affiliations:** Department of Chemistry Islamia University Bahawalpur Pakistan

## Abstract

Some novel symmetric 1,1′-dimethylferrocene derived amino acids have been prepared by the reaction of 1,1′-ferrocenedimethyldichloride with amino acids (glycine, alanine, phenylalanine and tyrosine). Their Cu(II), Co(II) and Ni(II) complexes, of the type [M(L)] where [M = Cu(II) and L = L^1^-L^5^] and [M(L)Cl_2_] where [M-Co(II)and Ni(II), L = L^1^-L^5^] have been prepared. The dicarboxylic acids and their metal complexes were characterized by their physical, analytical and spectral data. The [M(L)] complexes showed a square planar geometry whereas an octahedral geometry was observed for [M(L)Cl_2_] complexes. The title dicarboxylic acids and their metal complexes have also been screened for their antibacterial activity.


### SYMMETRIC 1,1′-DIMETHYLFERROCENE-DERIVED AMINO ACIDS: THEIR SYNTHESIS, CHARACTERIZATION, LIGATIONAL AND BIOLOGICAL PROPERTIES WITH Cu(II), Co(II) AND Ni(II) IONS.

Zahid H. Chohan

[1]Department of Chemistry, Islamia University, Bahawalpur (Pakistan)

### ABSTRACT

Some novel symmetric 1,1′-dimethylferrocene derived amino acids have been prepared by the reaction of 1,1′-ferrocenedimethyldichloride with amino acids (glycine, alanine, phenylalanine and tyrosine). Their Cu(II), Co(II) and Ni(II) complexes, of the type [M(L)] where [M=Cu(II) and $L=L^1$-$L^5$] and [$M(L)Cl_2$] where [M=Co(II)and Ni(II), $L=L^1$-$L^5$] have been prepared. The dicarboxylic acids and their metal complexes were characterized by their physical, analytical and spectral data. The [M(L)] complexes showed a square planar geometry whereas an octahedral geometry was observed for [$M(L)Cl_2$] complexes. The title dicarboxylic acids and their metal complexes have also been screened for their antibacterial activity.

### INTRODUCTION

There are significant evidences[1-4] that amino acid complexes are potentially used in the treatment of tumors. Various tumors tend to have poor blood supplies and therefore, amino acids have been effectively used to direct nitrogen mustards into the cancer cells. For example, phenylalanine mustard[5] is used in controlling malignant myeloma[6] and Burkitts' lymphoma[7] and, similarly sarcolysine[5] is used to treat wide range of tumors. Indeed, certain tumors and cancer cells are unable to produce all the amino acids synthesized by the normal cells. Therefore, these cells require an external supply of such essential amino acids to pass on to the cancer cells by the blood stream.

In the recent past a number of studies[8-12] have highlighted the utility of ferrocene and its derivatives in various applications[13-17]. Very few ferrocene-derived compounds have been used as ligands for the complex formation reactions. Keeping in view the significance of amino acids and their complexes as chemotheraptic agent and the chemistry of ferrocene or ferrocene-containing compounds as stable intermediates, a successful effort to join the chemistry of amino acids and ferrocene is made. For this purpose, some novel symmetric 1,1′-dimethylferrocene derived amino acids (Figure 1) have been synthesized and studied for their physicochemical, ligational behavior with Cu(II), Co(II) and Ni(II) metal ions and also for their antibacterial properties against bacterial strains, *Escherichia coli*, *Staphylococcus aureus*, *Pseudomonas aeruginosa* and *Klebsiella pneumonae.*

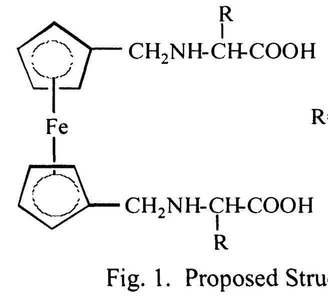

R= -H ($H_2L^1$), -$CH_3$($H_2L^2$), -$(CH_2)_2CH_3$($H_2L^3$), -$(CH_2)_3$Ph ($H_2L^4$) or -$(CH_2)_3$Ph-OH($H_2L^5$)

Fig. 1. Proposed Structure of the Ligands

### EXPERIMENTAL

Material and Methods

All solvents were used as Analar grade. 1,1′-Ferrocenedimethanol was obtained from Merck. 1,1′-Ferrocenedimethyldichloride derivative was prepared by a reported method[18] using thionyl chloride in triethylamine. All metals were used as chlorides. IR, $^1H$ NMR and $^{13}C$ NMR spectra were recorded on Philips Analytical PU 9800 FTIR and Brucker 250 MHz instruments. UV- Visible spectra were obtained on a Hitachi U-2000 double-beam spectrophotometer. Conductance of the metal complexes was determined in DMF at $10^{-3}$ dilution on a YSI-32 model conductometer. Magnetic measurements were done on solid complexes using the Gouy method. The synthesized dicarboxylic acids and their metal complexes were analyzed for C, H and N by Butterworth Laboratories Ltd. Melting points were recorded on a Gallenkamp apparatus and are uncorrected.

**Synthesis of Dicarboxylic acids**

A mixture of 1,1′-ferrocenedimethanol (0.65 g, 3.0 mmol), triethylamine (0.60 g, 6.0 mmol) and dichloromethane (20 mL) was cooled in an ice bath. Thionyl chloride (0.71 g, 6.0 mmol) in dichloromethane

(20 mL) was added into this mixture under $N_2$ at such a rate to keep the temperature between 15-20° C. After complete addition, the reaction mixture was kept at 20° C for 30 minutes and then stirred at 40° C for another 30 minutes. Ice was added and mixture stirred for another 5 minutes. A small amount of $NaHCO_3$ was then added to obtain pH 6.0. The organic layer was separated and dried over $CaCl_2$. Filtration and evaporation of the solvent gave a dark brown solid which was dissolved in dichloromethane (20 mL) and each amino acid (glycine, alanine, phenylalanine or tyrosine) (1.25 mmol) in dichloromethane (20 mL) was individually added to it. The reaction mixture was refluxed for 5 h under a slow stream of $N_2$. After allowing to cool to room temperature solvent was evaporated to give a yellow-orange solid which was recrystallized from chloroform.

**Synthesis of Metal Complexes**

Dicarboxylic acid (1.0 mmol) was dissolved in ethanol (30 mL) and warmed for several minutes. A solution of metal (II) chloride (1.0 mmol) in ethanol (20 mL) was added to the above solution. Then 2-3 drops of conc $H_2SO_4$ were added and mixture was refluxed for 3 h. During this time, precipitate was formed that was filtered, washed several times with warm ethanol and diethyl ether and, then dried over anhydrous $CaCl_2$.

**Antibacterial Studies**

The synthesized metal chelates in comparison to the ligands were screened for their antibacterial activity against pathogenic bacterial strains, *Escherichia coli, Staphylococcus aureus* and *Pseudomonas aeruginosa* and *Klebsiella pneumonae*. The paper disc diffusion method was adopted for the determination of antibacterial activity[19,20].

### RESULTS AND DISCUSSION

All the synthesized dicarboxylic acids (Fig 1) are soluble in polar solvents such as methanol, ethanol and acetonitrile, but are insoluble in weakly polar or non-polar solvents. All metal complexes dissolve only in DMF and DMSO. All of them are amorphous solid. Molar conductance values (14-18 $\Omega cm^2 mol^{-1}$) of the Cu(II) complexes in DMF show them to be non-electrolytes and the values (82-90 $\Omega cm^2 mol^{-1}$) for the Co(II) and Ni(II) complexes show all of them to be electrolytic in nature[21].

Table 1: Physical, Spectral and Analytical Data of the Dicarboxylic acids

| Dicarboxylic acid/ Mol. Form. | M.P. (°C) | IR ($cm^{-1}$) | Calc (Found %) C H N | Yield (%) |
|---|---|---|---|---|
| $H_2L^1$ $C_{16}H_{20}FeN_2O_4$ [359.85] | 178 | 3375 (s, NH), 1822 (s, COOH), 1553 (ms, C=C) | 53.4 5.6 7.8 (53.7) (5.5) (7.3) | 62 |
| $H_2L^2$ $C_{18}H_{26}FeN_2O_4$ [389.85] | 165 | 3370 (s, NH), 1825 (s, COOH), 1550 (ms, C=C) | 55.4 7.8 7.2 (55.3) (7.5) (7.0) | 60 |
| $H_2L^3$ $C_{22}H_{34}FeN_2O_4$ [445.85] | 170 | 3372 (s, NH), 1825 (s, COOH), 1552 (ms, C=C) | 59.2 7.6 6.3 (58.8) (7.5) (6.8) | 58 |
| $H_2L4$ $C_{34}H_{42}FeN_2O_4$ [597.85] | 182 | 3375 (s, NH), 1827 (s, COOH), 1550 (ms, C=C) | 68.2 7.0 4.7 (68.6) (6.9) (4.5) | 61 |
| $H_2L^5$ $C_{34}H_{42}FeN_2O_6$ [629.85] | 169 | 3410 (b, OH), 3380 (s, NH), 1830 (s, COOH), 1555 (ms, C=C) | 64.8 6.7 4.4 (65.1) (6.6) (4.6) | 59 |

**IR Spectra**

The important infrared frequencies of the uncomplexed dicarboxylic acids and its complexes along with their assignments are given in Tables 1 and 3, respectively. The IR spectra of the dicarboxylic acids show characteristic absorption bands at ~3372, ~1822 and ~1550 $cm^{-1}$ due to the ν(NH), ν(COOH) and ν(C=C) stretching vibrations[22] respectively. The bonding of the dicarboxylic acids to the metal atoms was investigated by comparing IR spectra of the free dicarboxylic acids with those of their metal complexes. The spectra of the complexes show significant changes as compared to that of the dicarboxylic acids. It can be seen that the bands due to the ν(NH) move towards lower frequency by 5-10 $cm^{-1}$ indicating their coordination to the metal atoms through the ν(NH) group. Deprotonation of the ν(COOH) was also indicated in the spectra of the complexes as the band due to the ν(COO) was observed at ~1575 $cm^{-1}$ which in turn, showed complexation through a deprotonated (COOH) group. Moreover, in the far IR region, three new bands around 365, 415, 450 $cm^{-1}$ assigned to the ν(M-Cl), ν(M-N) and ν(M-O) modes[23] respectively were found in the spectra of the Co(II) and Ni(II) complexes and not in the spectra of the dicarboxylic acids. The stretches due to the ν(M-N) and ν(M-O) were only found in the spectra of the Cu(II) complexes, however, bands due to the ν(M-Cl) in the far IR region were not found in the spectra of the Cu(II) complexes which indicated that the Co(II) and Ni(II) complexes possess an octahedral and the Cu(II) complexes a square planar geometry.

Table 2: Physical and Analytical Data of the Metal(II) Complexes

| Complex | Mol. Formula | M.P ($^{o}C$) (dec) | B.M ($\mu_{eff}$) | Calc (Found)% C H N |
|---|---|---|---|---|
| 1 | $C_{16}H_{18}CuFeN_2O_4$ [421.4] | 243-245 | 1.5 | 45.6 4.3 6.6 (45.3) (4.1) (6.8) |
| 2 | $C_{18}H_{24}CuFeN_2O_4$ [451.4] | 248-250 | 1.7 | 47.9 5.3 6.2 (48.1) (5.6) (6.3) |
| 3 | $C_{22}H_{32}CuFeN_2O_4$ [507.4] | 266-268 | 1.9 | 52.0 6.3 5.5 (52.4) (6.1) (5.5) |
| 4 | $C_{34}H_{40}CuFeN_2O_4$ [659.4] | 258-260 | 1.5 | 61.9 6.0 4.2 (62.2) (6.2) (4.0) |
| 5 | $C_{34}H_{40}CuFeN_2O_6$ [691.4] | 269-271 | 1.6 | 59.0 5.8 4.0 (59.3) (5.5) (3.8) |
| 6 | $C_{16}H_{18}Cl_2CoFeN_2O_4$ [487.68] | 258-260 | 4.3 | 39.4 3.7 5.7 (39.1) (3.5) (5.8) |
| 7 | $C_{18}H_{24}Cl_2CoFeN_2O_4$ [517.68] | 248-250 | 4.1 | 41.7 4.6 5.4 (41.5) (4.6) (5.1) |
| 8 | $C_{22}H_{32}Cl_2CoFeN_2O_4$ [573.68] | 263-265 | 4.3 | 46.0 5.5 4.8 (46.6) (5.4) (4.5) |
| 9 | $C_{34}H_{40}Cl_2CoFeN_2O_4$ [725.68] | 268-270 | 4.2 | 56.2 5.5 3.9 (56.3) (5.0) (4.1) |
| 10 | $C_{34}H_{40}Cl_2CoFeN_2O_6$ [757.68] | 258-260 | 4.2 | 53.9 5.3 3.7 (54.2) (5.2) (3.8) |
| 11 | $C_{16}H_{18}Cl_2NiFeN_2O_4$ [487.44] | 259-261 | 2.8 | 39.4 3.7 5.7 (39.2) (3.6) (5.5) |
| 12 | $C_{18}H_{24}Cl_2NiFeN_2O_4$ [517.44] | 262-264 | 3.0 | 41.7 4.6 5.4 (41.3) (4.7) (5.6) |
| 13 | $C_{22}H_{32}Cl_2NiFeN_2O_4$ [573.44] | 258-260 | 2.8 | 46.0 5.6 4.9 (46.1) (5.6) (5.1) |
| 14 | $C_{34}H_{40}Cl_2NiFeN_2O_4$ [775.44] | 260-262 | 2.9 | 56.2 5.5 3.9 (56.3) (5.4) (4.2) |
| 15 | $C_{34}H_{40}Cl_2NiFeN_2O_6$ [757.44] | 258-260 | 2.8 | 53.9 5.3 3.7 (54.1) (5.3) (3.9) |

**NMR Spectra**

The $^{1}H$ NMR and $^{13}C$ NMR spectra of the free dicarboxylic acids as well as some of their metal complexes, taken in DMSO-$d_6$ are listed in Table 4. The free dicarboxylic acids exhibited signals due to all the expected protons and carbons in their expected region and have been identified from the integration curve found to be equivalent to the total number of protons deduced from their proposed structures. These were identical to those reported[24-28] signals of the known compounds and therefore, gave further support for the compositions of these new dicarboxylic acids and their complexes as suggested by their IR and elemental analyses data. When these shifts were compared to those of the corresponding complexes, they exhibited a shift of some resonances. In each case, a broad singlet occurring downfield at $\delta$ 8.7-8.9 ppm assigned to (NH) undergoes a shift towards higher field by 0.15-0.2 ppm in the complexes. Also, the protons due to (COOH) found in the spectra of the dicarboxylic acids at $\delta$ 11.7-11.9 ppm suggested[29] the deprotonation of the carboxylic oxygen atom of the dicarboxylic acids on complexation. Similarly, all other protons and carbon resonances observed for the dicarboxylic acids shifted downfield in the spectra of their metal complexes thus showing the complexation phenomenon to occur.

The NMR spectra of the representative Cu(II) complexes with dicarboxylic acids ($L^1$-$L^5$) are only reported in Table 4. The spectra of other metal complexes showed similar characteristic features except the shift (0.5-1.5 ppm) of signals and therefore, are not included in Table 4.

**Electronic Spectra and Magnetic Moments**

The electronic spectra of the Cu(II) complexes showed two weak low-energy bands at 15150-16355 $cm^{-1}$ and 18770-19585 $cm^{-1}$ and a strong high-energy band at 30345-31770 $cm^{-1}$. The low-energy bands are in positions characteristic for a square planar configuration and may be assigned to $^2B_{1g} \rightarrow {}^2A_{1g}$ and $^2B_{1g} \rightarrow {}^2E_g$ transitions, respectively[30,31]. The strong high-energy band is assigned to metal $\rightarrow$ ligand charge transfer. Also, the magnetic moment values (1.5-1.9 B.M) for the Cu(II) complexes were found to be consistent with the proposed square planar structure (Fig 2A).

Table 3. Spectral Data of the Metal Complexes

| Complex | IR (cm $^{-1}$) | $\lambda_{max}$ (cm $^{-1}$) |
|---|---|---|
| 1 | 3370 (s, NH), 1575 (s, COO), 450 (m, M-O), 415 (m, M-N). | 30345, 18770, 16355 |
| 2 | 3375 (s, NH), 1570 (s, COO), 450 (m, M-O), 415 (m, M-N). | 31770, 19585, 15150 |
| 3 | 3370 (s, NH), 1575 (s, COO), 450 (m, M-O), 415 (m, M-N). | 30865, 19245, 15675 |
| 4 | 3370 (s, NH), 1575 (s, COO), 450 (m, M-O), 415 (m, M-N). | 30555, 18990, 15550 |
| 5 | 3370 (s, NH), 1575 (s, COO), 450 (m, M-O), 415 (m, M-N). | 30980, 19110, 16275 |
| 6 | 3375 (s, NH), 1570 (s, COO), 450 (m, M-O), 415 (m, M-N), 360 (m, M-Cl). | 20530, 16200, 7545 |
| 7 | 3370 (s, NH), 1575 (s, COO), 450 (m, M-O), 415 (m, M-N), 365 (m, M-Cl). | 19500, 17255, 8450 |
| 8 | 3375 (s, NH), 1570 (s, COO), 450 (m, M-O), 415 (m, M-N), 360 (m, M-Cl). | 20320, 17160, 7955 |
| 9 | 3370 (s, NH), 1575 (s, COO), 450 (m, M-O), 415 (m, M-N), 363 (m, M-Cl). | 20375, 16855, 8225 |
| 10 | 3375 (s, NH), 1575 (s, COO), 450 (m, M-O), 415 (m, M-N), 360 (m, M-Cl). | 19850, 16775, 8170 |
| 11 | 3370 (s, NH), 1570 (s, COO), 450 (m, M-O), 415 (m, M-N), 365 (m, M-Cl). | 25565, 15625, 9445 |
| 12 | 3370 (s, NH), 1575 (s, COO), 450 (m, M-O), 415 (m, M-N), 360 (m, M-Cl). | 26150, 15860,10315 |
| 13 | 3375 (s, NH), 1815 (s, COO), 450 (m, M-O), 415 (m, M-N), 360 (m, M-Cl). | 25775, 15775, 9880 |
| 14 | 3370 (s, NH), 1575 (s, COO), 450 (m, M-O), 415 (m, M-N), 360 (m, M-Cl). | 26055, 15880,10275 |
| 15 | 3370 (s, NH), 1575 (s, COO), 450 (m, M-O), 415 (m, M-N), 360 (m, M-Cl). | 25880, 15822,10110 |

s=strong, m=medium

The Co(II) complexes exhibit well resolved low-energy bands at 7545-8450, 16200-17255 and a strong high-energy band at 19500-20530 cm$^{-1}$ assigned to the transitions $^4T_{1g}(F) \rightarrow {}^4T_{2g}(F)$, $^4T_{1g}(F) \rightarrow {}^4A_{2g}(F)$ and $^4T_{1g}(F) \rightarrow {}^4T_{2g}(P)$ for high-spin octahedral geometry[32,33]. The magnetic susceptibility measurements (4.1-4.3 B.M) for Co(II) solid complexes are indicative of three unpaired electrons per Co(II) ion suggesting[34] an octahedral environment. The electronic spectra for the Ni(II) complexes show d-d bands in the region 25565-26150, 15675-15860 and 9445-10315 cm$^{-1}$. These are assigned to the transitions $^3A_{2g}(F) \rightarrow {}^3T_{2g}(F)$, $^3A_{2g}(F) \rightarrow {}^3T_{1g}(F)$ and $^3A_{2g}(F) \rightarrow {}^3T_{2g}(P)$ respectively, consistent with an octahedral configuration[35]. The magnetic measurements (2.8-3.0 B.M) were showed two unpaired electrons per Ni(II) ion suggesting[36] also an octahedral geometry for the Ni(II) complexes (Fig 2B).

Furthermore, a broad band centered at 22150-22500 cm$^{-1}$ observed for every complex was assigned[31] to the transition $^1A_{1g} \rightarrow {}^1E_{1g}$ in the iron atom of the ferrocenyl group indicate that there is no magnetic interaction between the Cu(II), Co(II) and Ni(II) ions and the diamagnetic Fe(II) ion.

Based on the above observations, it is proposed that the Cu(II) complexes have a square planar geometry (Fig 2A) whereas the Co(II) and Ni(II) complexes are octahedral (Fig 2B).

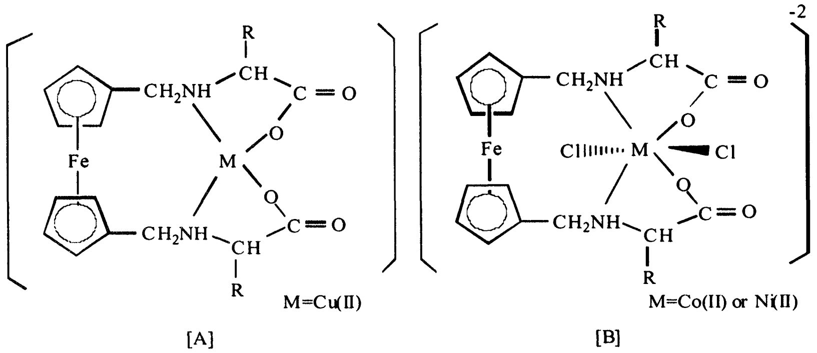

Fig. 2. Proposed Structure for the Cu(II), Co(II) and Ni(II) Complexes

**Antibacterial Properties**

The synthesized dicarboxylic acids and their metal complexes were evaluated for their antibacterial activity against *Escherichia coli (a), Pseudomonas aeruginosa (b), Staphylococcus aureus (c)* and *Klebsiella pneumonae (d)*. The compounds were tested at a concentration of 30 µg/0.01 mL in DMF solution using the paper disc diffusion method. The susceptibility zones measured in mm are reported in Table 5. The susceptibility zones were the clear zones around the discs. All the dicarboxylic acids were found to be biologically active and their metal complexes showed more significant antibacterial activity against one or more bacterial species in comparison to the uncomplexed ligands. In most of the cases chelation tends to make the dicarboxylic acids act as more powerful and potent bactericidal, thus killing more of the bacteria than the parent dicarboxylic acids. A possible explanation for the increased activity of the complexes is proposed. It may be suggested that in the chelated complex, the positive charge of the metal is partially shared with donor atoms and there is π-electron delocalization over the whole chelate ring. This increases the lipophilic character of the metal chelate and favors its permeation through lipoid layers of the bacterial membranes.

Table 4. $^1$H NMR and $^{13}$C NMR Data of Dicarboxylic acids and its Cu(II) Complexes

| Dicarboxylic acid/ Complex | $^1$H NMR (DMSO-d$_6$) (ppm) | $^{13}$C NMR (DMSO-d$_6$) (ppm) |
|---|---|---|
| $H_2L^1$ | 3.8 (t, 4H, ferrocenyl), 4.1 (t, 4H, ferrocenyl), 4.4 (t, 4H, $CH_2N$), 4.5-4.7 (m, 4H, $CH_2$), 8.7 (s, 2H, NH), 11.7 (s, 2H, COOH). | 38.1 (Fc.$CH_2$), 50.5 ($CH_2$), 68.7, 69.5, 83.7 (ferrocenyl), 176.8 (COOH). |
| $H_2L^2$ | 1.7 (s, 6H, CH3), 3.7 (t, 4H, ferrocenyl), 4.3 (t, 4H, ferrocenyl), 4.5 (t, 4H, $CH_2N$), 8.7 (s, 2H, NH), 11.7 (s, 2H, COOH). | 22.5 (CH3), 38.1 (Fc.$CH_2$), 54.8 (CH), 68.5, 69.5, 83.7 (ferrocenyl), 176.8 (COOH). |
| $H_2L^3$ | 1.6 (s, 6H, $CH_3$), 2.2-2.3 (m, 4H, $CH_2$), 2.4 (m, 4H, $CH_2$), 3.8 (t, 4H, ferrocenyl $CH_2$), 4.1 (t, 4H, ferrocenyl), 4.5 (t, 4H, $CH_2N$), 8.8 (s, 2H, NH), 11.8 (s, 2H, COOH). | 22.5 ($CH_3$), 38.2 (Fc.$CH_2$), 43.1, 42.4 ($CH_2$), 54.9 (CH), 68.7, 69.6, 83.7 (ferrocenyl), 176.8 (COOH). |
| $H_2L^4$ | 2.1 (dd, 4H, $CH_2$), 2.3-2.5 (m, 4H, $CH_2$), 2.5 (m, 4H, $CH_2$), 3.7 (t, 4H, ferrocenyl), 4.2 (t, 2H, ferrocenyl), 4.5 (t, 4H, $CH_2N$), 8.8 (s, 2H, NH), 7.2-7.3 (m, 6H, *m*-H+ *p*-H Ph), 7.4-7.5 (m, 4H, *o*-H Ph), 11.8 (s, 2H, COOH). | 38.1 (Fc.$CH_2$), 43.2, 42.4, 42.8 ($CH_2$), 54.9 (CH), 68.7, 69.5, 83.7 (ferrocenyl), 129.0, 130.7, 134.1 (Ph), 176.7 (COOH). |
| $H_2L^5$ | 2.2 (dd, 4H, $CH_2$), 2.4-2.5 (m, 4H, $CH_2$), 2.6-2.8 (m, 4H, $CH_2$), 3.8 (t, 4H, ferrocenyl), 4.2 (t, 2H, ferrocenyl), 4.6 (t, 4H, $CH_2N$), 7.3-7.4 (m, 6H, *m*-H+*p*-H Ph), 7.5-7.7 (m, 4H, *o*-H Ph), 8.9 (s, 2H, NH), 9.5 (s, 2H, OH), 11.9 (s, 2H, COOH). | 38.1 (Fc.$CH_2$), 43.2, 42.4, 42.8 ($CH_2$), 54.9 (CH), 68.5, 69.5, 83.7 (ferrocenyl), 129.1, 130.8, 134.5, 138.2 (Ph), 176.8 (COOH). |
| 1 | 3.9 (t, 4H, ferrocenyl), 4.2 (t, 4H, ferrocenyl), 4.6 (t, 4H, $CH_2N$), 4.6-4.8 (m, 4H, $CH_2$), 8.6 (s, 2H, NH). | 38.3 (Fc.$CH_2$), 50.6 ($NCH_2$), 68.8, 69.5, 83.8 (ferrocenyl) 173.5 (COO). |
| 2 | 1.8 (s, 6H, CH3), 3.8 (t, 4H, ferrocenyl), 4.5 (t, 4H, ferrocenyl), 4.6 (t, 4H, $CH_2N$), 4.7-4.8 (m, 4H, $CH_2$), 8.5 (s, 2H, NH). | 22.7 ($CH_3$), 38.2 (Fc.$CH_2$), 50.6 (CH), 68.8, 69.6, 83.8 (ferrocenyl). 173.7 (COO). |
| 3 | 1.8 (s, 6H, $CH_3$), 2.3-2.4 (m, 4H, $CH_2$), 2.6-2.8 (m, 4H, $CH_2$), 3.9 (t, 4H, ferrocenyl $CH_2$), 4.3 (t, 4H, ferrocenyl), 4.7 (t, 4H, $CH_2N$), 8.5 (s, 2H, NH). | 22.8 ($CH_3$), 38.4 (Fc.$CH_2$), 43.2, 42.5 ($CH_2$), 54.9 (CH), 68.8, 69.8, 83.9 (ferrocenyl), 173.6 (COO). |
| 4 | 2.3 (dd, 4H, $CH_2$), 2.4-2.5 (m, 4H, $CH_2$), 2.6-2.7 (m, 4H, $CH_2$), 3.9 (t, 4H, ferrocenyl), 4.3 (t, 2H, ferrocenyl), 4.6 (t, 4H, $CH_2N$), 8.6 (s, 2H, NH), 7.3-7.4 (m, 6H, *m*-H+ *p*-H Ph), 7.6-7.9 (m, 4H, *o*-H Ph). | 38.3 (Fc.$CH_2$), 43.3, 42.4, 42.9 ($CH_2$), 54.9 (CH), 68.8, 69.7, 83.8 (ferrocenyl), 129.1, 130.9, 134.3 (Ph), 173.7 (COO). |
| 5 | 2.3 (dd, 4H, $CH_2$), 2.5-2.6(m, 4H, $CH_2$), 2.8-2.9 (m, 4H, $CH_2$), 3.9 (t, 4H, ferrocenyl), 4.3 (t, 2H, ferrocenyl), 4.8 (t, 4H, $CH_2N$), 7.4-7.5 (m, 6H, *m*-H+*p*-H Ph), 7.7-7.8 (m, 4H, *o*-H Ph), 8.5 (s, 2H, NH), 9.8 (s, 2H, OH). | 38.2 (Fc.$CH_2$), 43.4, 42.6, 42.9 ($CH_2$), 54.9 (CH), 68.7, 69.6, 83.9 (ferrocenyl), 129.2, 130.9, 134.7, 138.4 (Ph), 173.8 (COO). |
| 6 | 4.0 (t, 4H, ferrocenyl), 4.3 (t, 4H, ferrocenyl), 4.8 (t, 4H, $CH_2N$), 4.5-4.8 (m, 4H, $CH_2$), 8.7 (s, 2H, NH). | 38.5 (Fc.$CH_2$), 50.7 ($NCH_2$), 68.9, 69.7, 83.7 (ferrocenyl) 173.6 (COO). |
| 7 | 1.9 (s, 6H, CH3), 4.0 (t, 4H, ferrocenyl), 4.4 (t, 4H, ferrocenyl), 4.8 (t, 4H, $CH_2N$), 4.8-5.0 (m, 4H, $CH_2$), 8.7 (s, 2H, NH). | 22.9 ($CH_3$), 38.4 (Fc.$CH_2$), 50.7 (CH), 68.9, 69.8, 83.8 (ferrocenyl). 173.8 (COO). |
| 8 | 2.0 (s, 6H, $CH_3$), 2.4-2.6 (m, 4H, $CH_2$), 2.8-2.9 (m, 4H, $CH_2$), 3.9 (t, 4H, ferrocenyl $CH_2$), 4.5 (t, 4H, ferrocenyl), 4.8 (t, 4H, $CH_2N$), 8.7 (s, 2H, NH). | 22.9 ($CH_3$), 38.6 (Fc.$CH_2$), 43.3, 42.7 ($CH_2$), 54.9 (CH), 69.1, 69.9, 83.9 (ferrocenyl), 173.7 (COO). |
| 9 | 2.4 (dd, 4H, $CH_2$), 2.6-2.9 (m, 4H, $CH_2$), 3.0-3.2 (m, 4H, $CH_2$), 3.9 (t, 4H, ferrocenyl), 4.5 (t, 2H, ferrocenyl), 4.8 (t, 4H, $CH_2N$), 8.7 (s, 2H, NH), 7.4-7.6 (m, 6H, *m*-H+ *p*-H Ph), 7.9-8.1 (m, 4H, *o*-H Ph). | 38.4 (Fc.$CH_2$), 43.6, 42.5, 43.0 ($CH_2$), 55.0 (CH), 68.8, 69.8, 83.9 (ferrocenyl), 129.2, 130.0, 134.5 (Ph), 173.9 (COO). |

| | | |
|---|---|---|
| 10 | 2.4 (dd, 4H, $CH_2$), 2.6-2.9(m, 4H, $CH_2$), 3.1-3.2 (m, 4H, $CH_2$), 4.0 (t, 4H, ferrocenyl), 4.4 (t, 2H, ferrocenyl), 4.9 (t, 4H, $CH_2N$), 7.5-7.6 (m, 6H, *m*-H+*p*-H Ph), 7.8-8.0 (m, 4H, *o*-H Ph), 8.6 (s, 2H, NH), 9.9 (s, 2H, OH). | 38.4 (Fc.$CH_2$), 43.6, 42.8, 42.9 ($CH_2$), 54.9 (CH), 68.8, 69.7, 83.9 (ferrocenyl), 129.3, 130.9, 134.9, 138.5 (Ph), 173.9 (COO). |
| 11 | 4.1 (t, 4H, ferrocenyl), 4.4 (t, 4H, ferrocenyl), 4.7 (t, 4H, $CH_2N$), 4.8-4.9 (m, 4H, $CH_2$), 8.7 (s, 2H, NH). | 38.3 (Fc.$CH_2$), 50.6 ($NCH_2$), 68.8, 69.5, 83.8 (ferrocenyl) 173.5 (COO). |
| 12 | 1.9 (s, 6H, CH3), 4.0 (t, 4H, ferrocenyl), 4.4 (t, 4H, ferrocenyl), 4.3 (t, 4H, $CH_2N$), 4.5-4.7 (m, 4H, $CH_2$), 8.2 (s, 2H, NH). | 22.7 ($CH_3$), 38.2 (Fc.$CH_2$), 50.6 (CH), 68.8, 69.6, 83.8 (ferrocenyl). 173.7 (COO). |
| 13 | 1.5 (s, 6H, $CH_3$), 2.1-2.3 (m, 4H, $CH_2$), 2.7-2.9 (m, 4H, $CH_2$), 3.5 (t, 4H, ferrocenyl $CH_2$), 4.0 (t, 4H, ferrocenyl), 4.8 (t, 4H, $CH_2N$), 8.2 (s, 2H, NH). | 22.8 ($CH_3$), 38.4 (Fc.$CH_2$), 43.2, 42.5 ($CH_2$), 54.9 (CH), 68.8, 69.8, 83.9 (ferrocenyl), 173.6 (COO). |
| 14 | 2.1 (dd, 4H, $CH_2$), 2.3-2.5 (m, 4H, $CH_2$), 2.7-2.9 (m, 4H, $CH_2$), 3.9 (t, 4H, ferrocenyl), 4.1 (t, 2H, ferrocenyl), 4.5 (t, 4H, $CH_2N$), 8.7 (s, 2H, NH), 7.1-7.3 (m, 6H, *m*-H+ *p*-H Ph), 7.7-7.9 (m, 4H, *o*-H Ph). | 38.3 (Fc.$CH_2$), 43.3, 42.4, 42.9 ($CH_2$), 54.9 (CH), 68.8, 69.7, 83.8 (ferrocenyl), 129.1, 130.9, 134.3 (Ph), 173.7 (COO). |
| 15 | 2.0 (dd, 4H, $CH_2$), 2.2-2.4(m, 4H, $CH_2$), 2.8-2.9 (m, 4H, $CH_2$), 3.8 (t, 4H, ferrocenyl), 4.2 (t, 2H, ferrocenyl), 4.6 (t, 4H, $CH_2N$), 7.2-7.5 (m, 6H, *m*-H+*p*-H Ph), 7.8-7.9 (m, 4H, *o*-H Ph), 8.3 (s, 2H, NH), 9.9 (s, 2H, OH). | 38.2 (Fc.$CH_2$), 43.4, 42.6, 42.9 ($CH_2$), 54.9 (CH), 68.7, 69.6, 83.9 (ferrocenyl), 129.2, 130.9, 134.7, 138.4 (Ph), 173.8 (COO). |

Table 5. Antibacterial Activity Data

| Dicarboxylic acid/ Complex | Microbial Species a | b | c | d |
|---|---|---|---|---|
| $H_2L^1$ | ++ | + | + | ++ |
| $H_2L^2$ | ++ | + | ++ | + |
| $H_2L^3$ | ++ | + | + | ++ |
| $H_2L^4$ | + | ++ | - | + |
| $H_2L^5$ | + | + | + | ++ |
| 1 | +++ | ++ | +++ | +++ |
| 2 | +++ | +++ | ++ | ++ |
| 3 | +++ | ++ | +++ | ++++ |
| 4 | +++ | ++ | ++ | +++ |
| 5 | ++++ | +++ | +++ | +++ |
| 6 | +++ | +++ | +++ | ++ |
| 7 | ++++ | ++ | +++ | +++ |
| 8 | ++ | +++ | + | ++++ |
| 9 | +++ | +++ | +++ | ++ |
| 10 | +++ | +++ | +++ | +++ |
| 11 | +++ | +++ | ++ | +++ |
| 12 | +++ | ++++ | +++ | ++ |
| 13 | ++ | +++ | ++ | +++ |
| 14 | ++ | +++ | +++ | ++ |
| 15 | +++ | ++ | ++ | +++ |

a= *Escherichia coli*, b= *Staphylococcus aureus*,
c= *Pseudomonas aeruginosa* d= *Klebsiella pneumonae*
Inhibition zone diameter mm (% inhibition): +, 6-10 (27-45 %); ++, 10-14 (45-64 %); +++, 14-18 (64-82 %); ++++, 18-22 (82-100 %). Percent inhibition values are relative to inhibition zone (22 mm) of the most active compound with 100 % inhibition.

### ACKNOWLEDGEMENT

The author gratefully acknowledges the help of Department of Microbiology, Qaid-e-Azam Medical College, Bahawalpur, in undertaking the antibacterial studies.

### REFERENCES

1. S. E. Livingstone and I. D. Nolan, *Inorg. Chem*, **7**, 1447 (1968).
2. B. Rosenberg, *Plat. Met. Rev*, **15**, 42 (1971).
3. R. J. Bromfield, R. H. Dainty, R. D. Gillard and B. T. Heaton, *Nature*, **223**, 735 (1969).

4. D. R. Williams, *Inorg. Chim. Acta*, 123 (1972).
5. J. A. Stock, *Chem. Brit*, **6**, 11 (1970).
6. J. A. Stock, "*The Biology of Cancer*", Eds. E. J. Ambrose and F. J. C. Roe, Van Nostrand, London, Ch 9, (1966).
7. P. Clifford, S. Singh. J. Stjernsward and G. Klein, *Cancer. Res*, **27**, 2578 (1967).
8. E. I. Edwards, R. Epton and G. Marr, *J. Organomet. Chem*, **85**, C23 (1975).
9. B. W. Rockett, and G. Marr, *J. Organomet. Chem*, **123**, 123 (1976).
10. G. Huang, Y. Liang, and Y. Ma, *J. Coord. Chem*, **26**, 237 (1992).
11. R. Butler, "*Organometallic Chemistry, Specialist Periodical Reports*", Ed, E. W. Abel, , Vol 21, Chapter 14, Cambridge, (1992).
12. C. R. Kollmar, M. Couty and O. Kahn, *J. Am. Chem. Soc*, **113**, 7994.(1991).
13. E. C. Constable, *Angew. Chem. Int. Ed. Engl*, **30**, 407 (1991).
14. U. T. Muller-Westerhoff and B. Vance, *Coord. Chem. Rev*, **16**, 595 (1987).
15. M. L. H. Green, *Polyhedron*, **11**, 1489.(1992).
16. S. Ghosal, M. Samoe, P. N. Prasad and J. J. Tufariello, *J. Phys. Chem*, **94**, 2847 (1990).
17. P. D. Beer, L. E. Nation, S. W. L. McWinnie, M. E. Harman, M. B. Hursthouse, M. I. Ogden and A. H White, *J. Chem. Soc (Dalton Trans)*, 2485 (1991).
18. S. Gronowitz, and S. Liljefors, *Chim. Scripta*, **13**, 39 (1979).
19. Z. H. Chohan and H. Pervez, *Synth. React. Inorg. Met-Org. Chem*, **23**, 1061 (1993).
20. Z. H. Chohan and *S. K. A. Sherazi, , Synth. React. Inorg. Met-Org. Chem*, **29**, 105 (1999).
21. W. J. Geary, *Coord. Chem. Rev*, **7**, 81 (1971).
22. J. E. Kovacic, *Spectrochim. Acta*, **23A**, 183 (1967).
23. K. Nakamoto, "*Infrared Spectra of Inorganic and Coordination Compounds*", J. Wiley, New York, (1963).
24. D. H. Williams and I. Fleming, "*Spectroscopic Methods in Organic Chemistry*", 4th Ed, Mc Graw Hill, London, (1989).
25. Z. H. Chohan, "*Synthesis of Heterocylic Compounds via Ferrocene*" M.Sc Thesis, University Of Strathclyde, U.K, (1983).
26. Z. H. Chohan and M. Praveen, *Metal-Based Drugs*, **6**, 149 (1999).
27. M. A. Metwally, E. E. M. Kandel and F. A. Amer, *J. Ind. Chem. Soc*, **LXIV**, 753 (1987).
28. S. B. Wilkes, I. R. Butler, A. E. Underhill, M. B. Hursthouse, D. E. Hibbs and K. M. A. Malik, *J. Chem. Soc (Dalton Trans)*, 897 (1995).
29. G. Domazetis and M. F. Mackay, *J. Cryst. Mol. Struct*, **9**, 57 (1979).
30. B. N. Figgis, "*Introduction to Ligand Fields*", J. Wiley, New York, (1976).
31. A. B. P. Lever, "*Inorganic Electronic Spectroscopy*", Elsevier, Amsterdam, (1984).
32. A. D. Liehr, *J. Phys. Chem*, **67**, 1314 (1967).
33. R. L. Carlin,., "*Transition Metal Chemistry*", Ed, R. L. Carlin, Vol 1, Marcel Decker, New York, (1965).
34. D. W. Meek, R. S. Drago and T. S. Piper, *Inorg. Chem.*, **1**, 285 (1962).
35. R. S..Drago, "Physical Methods in Inorganic Chemistry", Reinhold, New York, (1965).
36. B. N. Figgis and J. Lewis, *Prog. Inorg. Chem.*, **6**, 87 (1964).

**Received: July 28, 2000- Accepted: September 11, 2000 -**
**Received in revised camera-ready format: September 15, 2000**

